# Detection of biomagnetic signals from induced pluripotent stem cell-derived cardiomyocytes using deep learning with simulation data

**DOI:** 10.1038/s41598-024-58010-0

**Published:** 2024-03-27

**Authors:** Takeshi Yamaguchi, Yoshiaki Adachi, Takashi Tanida, Katsutoshi Taguchi, Yoshinobu Oka, Takashi Yoshida, Wook-Cheol Kim, Kenji Takahashi, Masaki Tanaka

**Affiliations:** 1 Department of Anatomy and Neurobiology, Graduate School of Medical Science, Kyoto Prefectural University of Medicine, Kyoto, 602-8566 Japan; 2 Applied Electronics Laboratory, Kanazawa Institute of Technology, Ishikawa, 920-1331 Japan; 3 Department of Veterinary Anatomy, Graduate School of Veterinary Science, Osaka Metropolitan University, Osaka, 598-8531 Japan; 4 Department of Pediatric Orthopaedics, Graduate School of Medical Science, Kyoto Prefectural University of Medicine, Kyoto, 602-8566 Japan; 5 Department of Orthopaedic Surgery, North Medical Center, Kyoto Prefectural University of Medicine, Kyoto, 629-2261 Japan; 6 Department of Pediatric Orthopaedic Surgery and Ilizarov Center, Uji Takeda Hospital, Kyoto, 611-0021 Japan; 7 Department of Orthopaedics, Graduate School of Medical Science, Kyoto Prefectural University of Medicine, Kyoto, 602-8566 Japan

**Keywords:** Electrophysiology, Induced pluripotent stem cells, Machine learning, Computational models, Superconducting devices

## Abstract

The detection of spontaneous magnetic signals can be used for the non-invasive electrophysiological evaluation of induced pluripotent stem cell-derived cardiomyocytes (iPS-CMs). We report that deep learning with a dataset that combines magnetic signals estimated using numerical simulation and actual noise data is effective in the detection of weak biomagnetic signals. To verify the feasibility of this method, we measured artificially generated magnetic signals that mimic cellular magnetic fields using a superconducting quantum interference device and attempted peak detection using a long short-term memory network. We correctly detected 80.0% of the peaks and the method achieved superior detection performance compared with conventional methods. Next, we attempted peak detection for magnetic signals measured from mouse iPS-CMs. The number of detected peaks was consistent with the spontaneous beats counted using microscopic observation and the average peak waveform achieved good similarity with the prediction. We also observed the synchronization of peak positions between simultaneously measured field potentials and magnetic signals. Furthermore, the magnetic measurements of cell samples treated with isoproterenol showed potential for the detection of chronotropic effects. These results suggest that the proposed method is effective and has potential application in the safety assessment of regenerative medicine and drug screening.

## Introduction

In recent years, induced pluripotent stem cell-derived cardiomyocytes (iPS-CMs) have attracted attention as cell materials in regenerative medicine^1–3^. For example, regenerative therapies are rapidly developing, such as the transplantation of cardiac cell sheets created from human iPS cells for patients with myocardial infarction or dilated cardiomyopathy^4,5^. Additionally, in the field of drug discovery research, screening using iPS-CMs is expected to be used as a method for the evaluation of the safety and effectiveness of drug candidates^6,7^. For such practical applications, rapid and automatable cell safety assessment is mandatory. As a quality evaluation for regenerative medical materials, it is necessary to determine the degree of cell differentiation and maturity. In drug screening, it is required to detect abnormalities or changes in the beating rhythm of iPS-CMs, such as proarrhythmia. The field potential (FP) measurement^8^ and membrane potential-sensitive fluorescent dye^7^ are commonly used as evaluation methods for the electrophysiological properties of iPS-CMs. However, the problems that remain are that they are invasive or performed under non-physiological conditions^9^.

Synchronized electrical activity in a cell population produces intracellular and extracellular currents, which generate weak magnetic fields^10,11^. The magnetic fields of a propagating action potential (AP) reflect the electrophysiological properties of cells, and their rhythm and variability can be regarded as indicators of cell quality and activity. We focus on the merits: magnetic measurement is non-invasive, non-contact, and non-destructive. Magnetic measurement has the advantage that samples can continue to be cultured and observed after assessment. By contrast, researchers ^10,11^ have measured magnetic signals from cultured cells in only a few studies and measurement remains challenging. A highly sensitive magnetometer is required to detect weak magnetic signals from cultured cells. The most sensitive magnetometer in practical use is a superconducting quantum interference device (SQUID) magnetometer. In this study, we used the SQUID magnetometer developed by our group^12–15^.

Using numerical simulation, we preliminarily estimated that magnetic signals from mouse iPS-CMs are of the same order of intensity as background noise in the measurement environment. Therefore, it is not easy to detect these signals using conventional methods such as visual inspection, frequency analysis, and pattern matching. We analyzed the measured data using a fast Fourier transform and autoregressive model, but we found no spectra that could be considered magnetic signals originating from the cells. Deep learning has been used in electrocardiogram (ECG) analysis for rhythm classification, arrhythmia detection, and filtering out noisy data^16–19^. Researchers have also reported that deep learning effectively distinguishes between drug-free and drugged categories based on AP^20^ and predicts arrhythmia from Ca^2+^ cycling^9^. In particular, a long short-term memory (LSTM) network is excellent in the classification of time series data^20^. Therefore, we hypothesized that LSTM networks could effectively detect weak magnetic signals from iPS-CMs and validated this hypothesis. In this study, we aimed to evaluate the rhythm of cellular electrical activity and its changes by detecting peak regions from the magnetic measured data through an LSTM network classification task. Because there is no public database for cellular magnetic signals like PhysioNet for ECG^21^, and it is not easy to identify peak regions manually, preparing a correctly labeled dataset is an important step. We adopted the approach to train LSTM networks using newly generated data by superimposing magnetic signals from iPS-CMs estimated using numerical simulation on the actual background noise measured by the SQUID magnetometer.

To verify the feasibility of this method, we artificially generated magnetic signals that mimic cellular magnetic fields and measured them. When we attempted peak detection using a trained network, we correctly detected 80.0% of the peaks. We also examined the effect of frequency range and noise data on the detection results. Additionally, a comparison with the existing peak detection method, the scaled template technique^22^, demonstrated that deep learning was superior in detection capability. We then measured magnetic signals from mouse iPS-CMs on days 19–21 of differentiation. The number of detected peaks was in good agreement with the spontaneous beats counted using microscopic observation. The simultaneous measurement data of magnetic signals and FPs showed the synchronization of peak positions. Finally, we measured cell samples before and after administering a chronotropic drug, isoproterenol, and obtained results that suggested the detectability of beating rate changes.

## Results

### Overview of peak region detection for magnetic signals from iPS-CMs using deep learning

**Figure 1:**
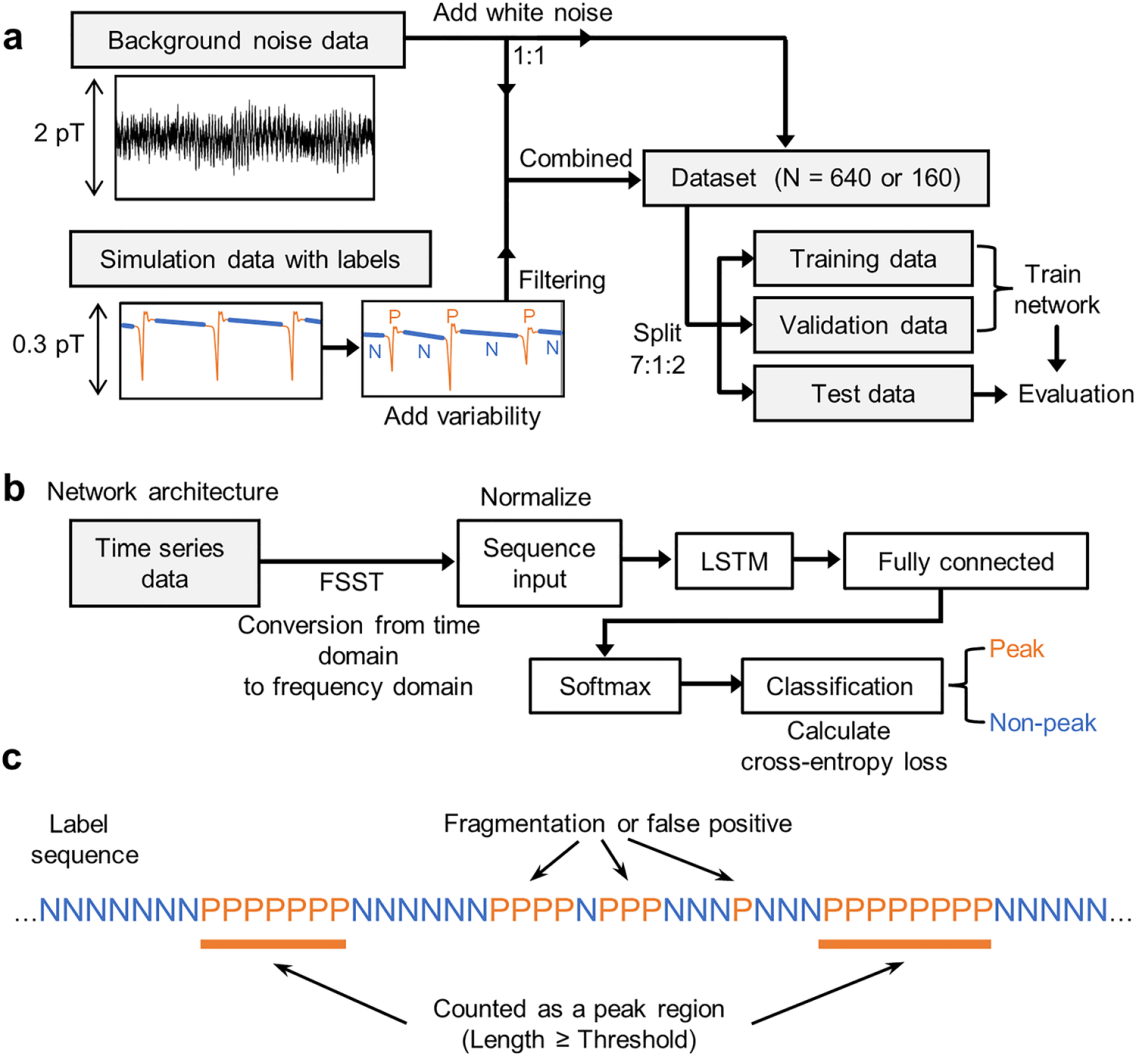
Overview of peak region detection for magnetic signals from iPS-CMs using deep learning. (**a**) Block diagram of dataset preparation. We prepared data that contained the magnetic signals estimated using numerical simulation combined with background noise. We divided them into training, validation, and test data in a ratio of 7:1:2 and used them to train a network. (**b**) Network architecture. The FSST converted the time series data to the frequency domain data. After normalization, the data were passed from a sequence input layer to an LSTM layer. A fully connected layer with an output size of two equal to the number of classes was followed by softmax and classification layers. (**c**) Determination of peak regions from output label sequences. The label specifies whether each data point was of the peak (label 'P') or non-peak (label 'N') class. The segments labeled class P that were longer than the set threshold were identified as peak regions.

We aimed to detect peaks for magnetic signals measured from iPS-CMs using deep learning. The flow of peak region detection is as follows: The magnetic signals from iPS-CMs estimated using simulation and background noise data were combined (added together) to prepare a dataset for network training (Fig. 1a). We defined two classes, peak (P) and non-peak (N), and classified each data point into one of the classes. A schematic diagram of the network used to classify time series data into two classes is shown in Fig. 1b. The criteria for determining peak regions from output labels are shown in Fig. 1c.

### Optimization of AP models

**Figure 2:**
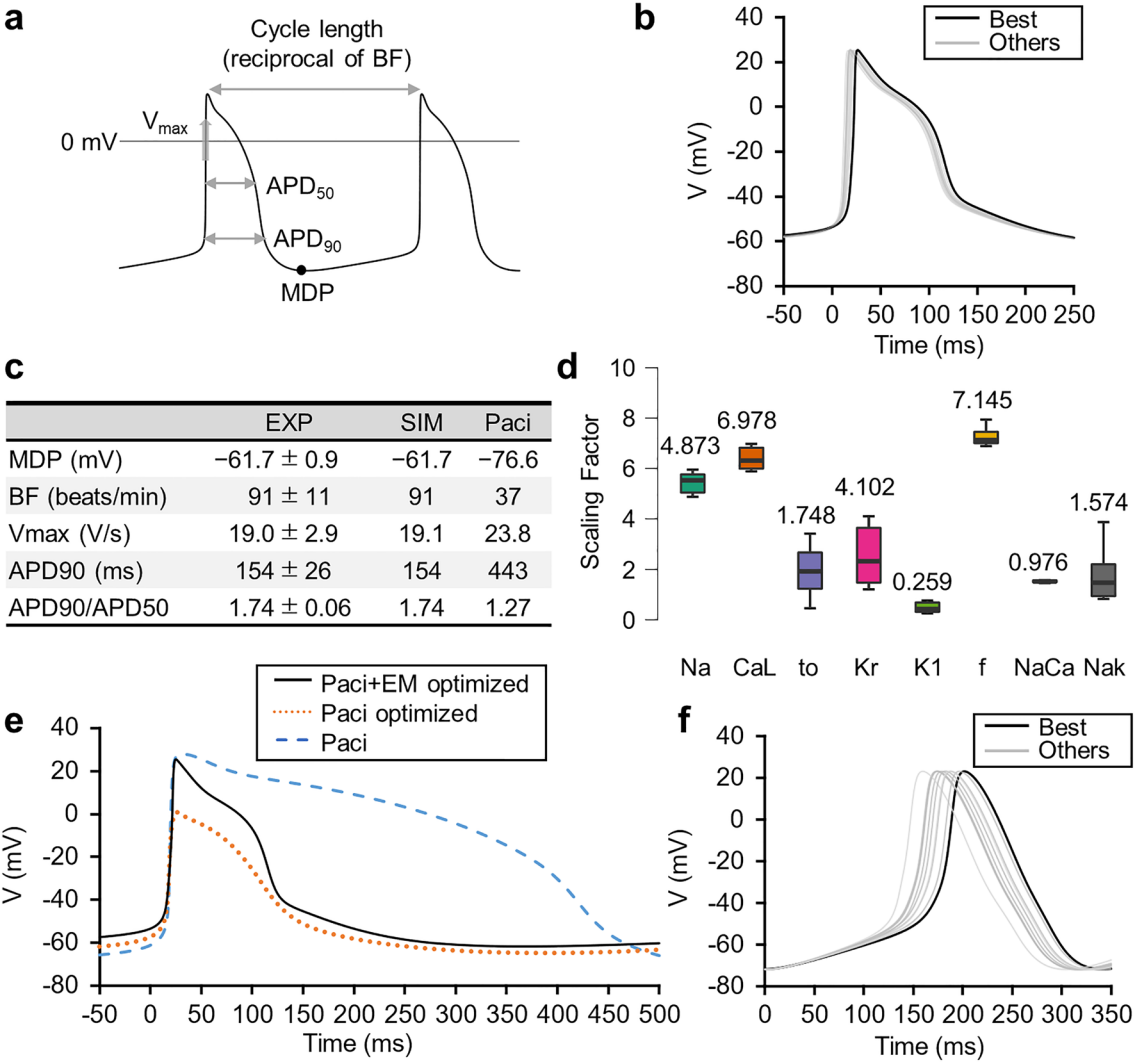
Optimization of AP models. (**a**) Five parameters characterizing AP: maximum diastolic potential (MDP), maximum upstroke velocity (Vmax), AP duration of 90% repolarization (APD90), APD90/50, and beat frequency (BF). (**b**) APs calculated from the optimized models for ten independent GAs are overlaid. The solid black line indicates the best model and the solid gray line indicates the other nine. All APs are shown to be aligned with the rise timing as 0 ms. (**c**) Comparison of the AP parameters. Experimental values of mouse iPS-CMs, values calculated using the proposed model, and values calculated using the original Paci model. (**d**) Optimized conductances are shown as scaling factors for the base model. We plotted the scaling factors of the optimized models for the ten independent GAs. The numbers in this panel are the conductances of the best model. Na: fast sodium current, CaL: L-type calcium current, to: transient outward current, Kr: rapid delayed rectifier current, K1: inward rectifier current, f: hyperpolarization-activated cyclic-nucleotide-gated funny current, NaCa: sodium-calcium exchanger, and NaK: sodium–potassium pump. (**e**) Overlay plot of AP waveforms calculated using the original Paci model (blue dashed line), optimized Paci model after replacing* I*_f_ with that of the mouse embryonic cardiomyocyte model (black solid line), and optimized Paci model (red dotted line). (**f**) Pacemaker-like cell AP model optimized to reproduce the experimental results. The APs calculated from the optimized models for the ten independent GAs are overlaid. The solid black line indicates the best model and the solid gray line indicates the other nine. All APs are shown to be aligned with MDP as 0 ms.

As a preliminary step to estimate magnetic signals from mouse iPS-CMs, we created an AP model of ventricular-type mouse iPS-CMs based on the human iPS-CMs model (Paci model^23^). We optimized the six ion channel conductances and the maximum current values of the sodium-calcium exchanger and sodium–potassium pump, a total of eight parameters, to reproduce the experimental results using the patch-clamp technique^24^. We performed the optimization using a genetic algorithm (GA)^25^, which heuristically finds an approximate solution by mimicking biological evolution (Supplementary Fig. S1). To evaluate each model in the population, we defined a score from five parameters characterizing AP (Fig. 2a) as follows:

1 $$\begin{array}{*{20}c} {Score = \sum \left( {\frac{{APParameter_{model} - APParameter_{experiment} }}{{SE_{experiment} }}} \right)^{2} } \\ \end{array}$$

We decided that the closer this score was to zero, the better the model reproduced the experimental results. We repeated the GAs ten times independently and adopted the model with the best score in this study. Considering the significant difference in beating rate between mice and humans, we replaced the current of *I*_f_ (funny current), which is involved in the spontaneous beating of cardiomyocytes^26^, with that of the mouse embryonic cardiomyocyte AP model^27^. As a result, the APs computed by the optimized models for each of the ten independent GAs converged to nearly the same waveform and the five AP parameters were consistent with the experimental values (Fig. 2b, c). The scaling factors of conductances relative to the base model are shown in Fig. 2d. The comparison of AP waveforms calculated by the original Paci model, the optimized Paci model (*I*_f_ was not replaced), and the adopted model is shown in Fig. 2e.

We also created an AP model of pacemaker-like cells differentiated from mouse iPS cells based on the AP model of the sinus node (YNI model^28^). We optimized the scaling factors for the five conductances using the GAs to reproduce the three AP parameters (MDP, APD90, and Peak) described in previous studies^29^. The APs computed with the optimized model are shown in Fig. 2f and the scaling factors of the conductances are shown in Supplementary Table S1.

### Two-dimensional simulation of the electrical activity of iPS-CMs

**Figure 3:**
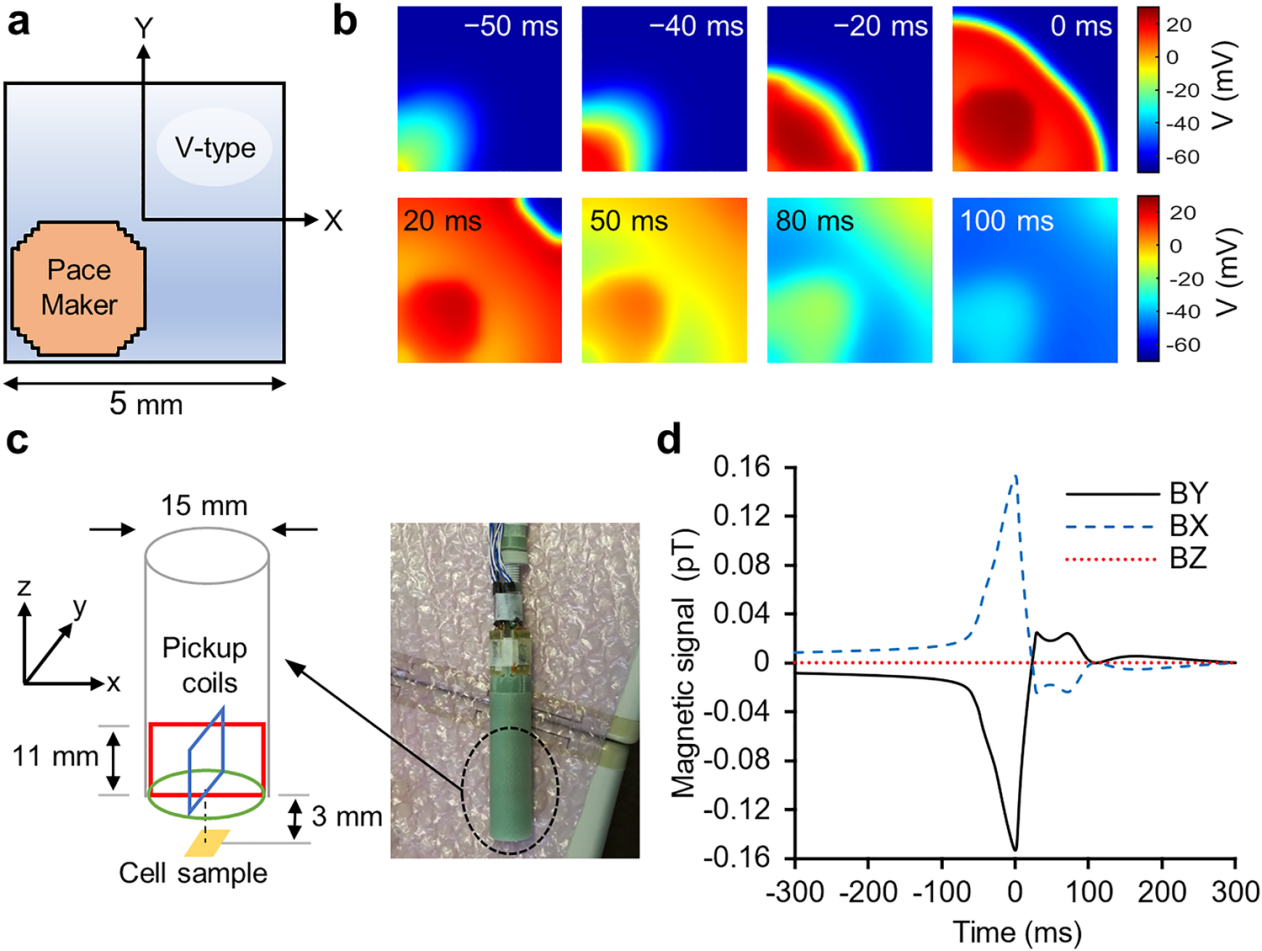
2D simulation of the electrical activities of iPS-CMs. (**a**) Cell distribution used in the numerical simulation. The space was discretized with Δ*x* = Δ*y* = 60 μm, and 84 × 84 units were used in the calculation. Pacemaker-like cells were placed in the lower left corner as a cluster of 1440 units, corresponding to 20% of all units. (**b**) Snapshots of the AP map. The time shown in each snapshot shows the relative time when the peak of the magnetic signal is defined as 0 ms. (**c**) Assumed dimensions of the magnetometer and its virtual position relative to a sample. The photo on the right shows the actual vector-type SQUID magnetometer. (**d**) Calculated waveforms of the three perpendicular components of the magnetic field (60 s after the initial conditions). The blue dashed line, black solid line, and red dotted line represent the magnetic field's *x*, *y*, and *z* components, respectively.

To estimate the magnetic signals generated by cultured iPS-CMs, we performed a two-dimensional (2D) electrical activity simulation of the cell population using the two AP models optimized in the previous section. We performed the calculations using geometry based on the measured size of the actual cell samples (Fig. 3a). As reported in published studies^3,30^, we set the cell number ratio between the two cell types to 80:20. We based the placement on the observation that pacemaker-like cells are distributed in clusters^31^. We modeled a 2D sheet of cardiac cells using the following partial differential equation^32^:

2 $$\begin{array}{*{20}c} {\frac{\partial V}{{\partial t}} = - \frac{{I_{ion} }}{{C_{m} }} + \frac{1}{{{\uprho }_{{\text{x}}} {\text{S}}_{{\text{x}}} C_{m} }}\frac{{\partial^{2} V}}{{\partial x^{2} }} + \frac{1}{{{\uprho }_{{\text{y}}} {\text{S}}_{{\text{y}}} C_{m} }}\frac{{\partial^{2} V}}{{\partial y^{2} }}} \\ \end{array}$$

where *V* is the membrane potential, *I*_ion_ is the total membrane current,* C*_m_ is the cell capacitance per unit area, ρ is the averaged cellular resistivity, and S is the surface-to-volume ratio. Assuming the isotropy of cardiomyocytes, we set ρ_x_ = ρ_y_ and S_x_ = S_y_. The time variation of the AP map is shown in Fig. 3b. We observed that excitation propagated from the cluster of pacemaker-like cells to the ventricular-type cardiomyocytes.

We calculated the magnetic fields generated from the intracellular flow currents. We set the virtual positional relationship between the pickup coils in the three axial directions and a sample to be equivalent to that of the actual measurement (Fig. 3c). The calculated waveforms are shown in Fig. 3d. The polarities of the waveforms in the *x*-axis direction and *y*-axis direction were opposite to each other, and the peak amplitude was 0.153 pT. The signal intensity in the *z*-axis direction was almost zero because the magnetic fields penetrating the coil upward and downward in the *z*-axis direction were approximately the same strength and canceled each other out. We estimated the cycle length to be 1050 ms.

### Preparation of the training dataset

The following procedures were used to add variability to the dataset (Fig. 1a). The peak waveforms estimated using simulation were stretched to random time lengths based on a normal distribution. The magnitude of variation (coefficient of variation = 0.21) was determined from the measured FP data of iPS-CMs (Supplementary Fig. S2). Amplitudes were also randomly varied in the 0.5–2.0 fold range. Background noise data were obtained by measuring the magnetic field inside the shielded box with no sample using the SQUID magnetometer. Before both data were combined, the simulation data were filtered through high-pass filters (HPF) at 3 Hz, low-pass filters (LPF) at 100 Hz, and notch filters at 60 Hz to be consistent with the noise data. An equal number of combined and noise-only data were prepared, and Gaussian noise with a standard deviation of 0.01 pT was added for data augmentation. The label data paired with the waveform data specified whether each data point was a peak (label ‘P’) or non-peak (label ‘N’) class. The dataset was split into 70% training, 10% validation, and 20% test datasets.

### Architecture of the network used for classification

The network architecture (Fig. 1b) started with a sequence input layer. Instead of raw waveforms, the frequency spectrum of each data point was calculated using a Fourier synchro-squeezed transform (FSST)^33^ and used as input for training. An LSTM layer was placed next to the input layer. The LSTM layer can learn the long-term dependencies of the input data^16^. The number of hidden units adjusts the information stored between each time step. Too many hidden units can cause overfitting^34^ and also have a significant impact on the computation time. The number of hidden units was determined to be 400 to balance the computation time and classification accuracy (Supplementary Fig. S3). Following the LSTM layer, a fully connected layer was implemented. It took the features extracted by the LSTM layer, multiplied them by the weight matrix, and added the bias vector. The softmax function was then used as the activation function in a softmax layer, yielding output values between 0 and 1: the predicted probability of classes. Finally, the cross-entropy loss was computed in a classification layer based on the output from the softmax layer. The adaptive moment estimation algorithm^35^, which minimizes the loss function, was used to optimize each weight matrix and bias vector. From the output labels, the segments labeled class P that were longer than the set threshold were identified as peak regions (Fig. 1c). This selection eliminated false positives and avoided one peak region being erroneously overcounted because of fragmentation.

### Measurement of the background noise

Before the main experiment, the background noise inside the mu-metal magnetically shielded box (MSB) was measured using the SQUID magnetometer. The standard deviation of the magnetic flux density for 60 s was defined as the noise level. As a result, the noise level was 0.218 pT in the *x*-axis direction and 0.146 pT in the *y*-axis direction. Because the estimated amplitude of the magnetic signal from iPS-CMs was 0.153 pT, as shown in the previous section, the *y* component of the magnetic field was targeted for peak region detection in the experiments with cell samples.

### Validation of the proposed method using artificially generated magnetic signals

**Figure 4:**
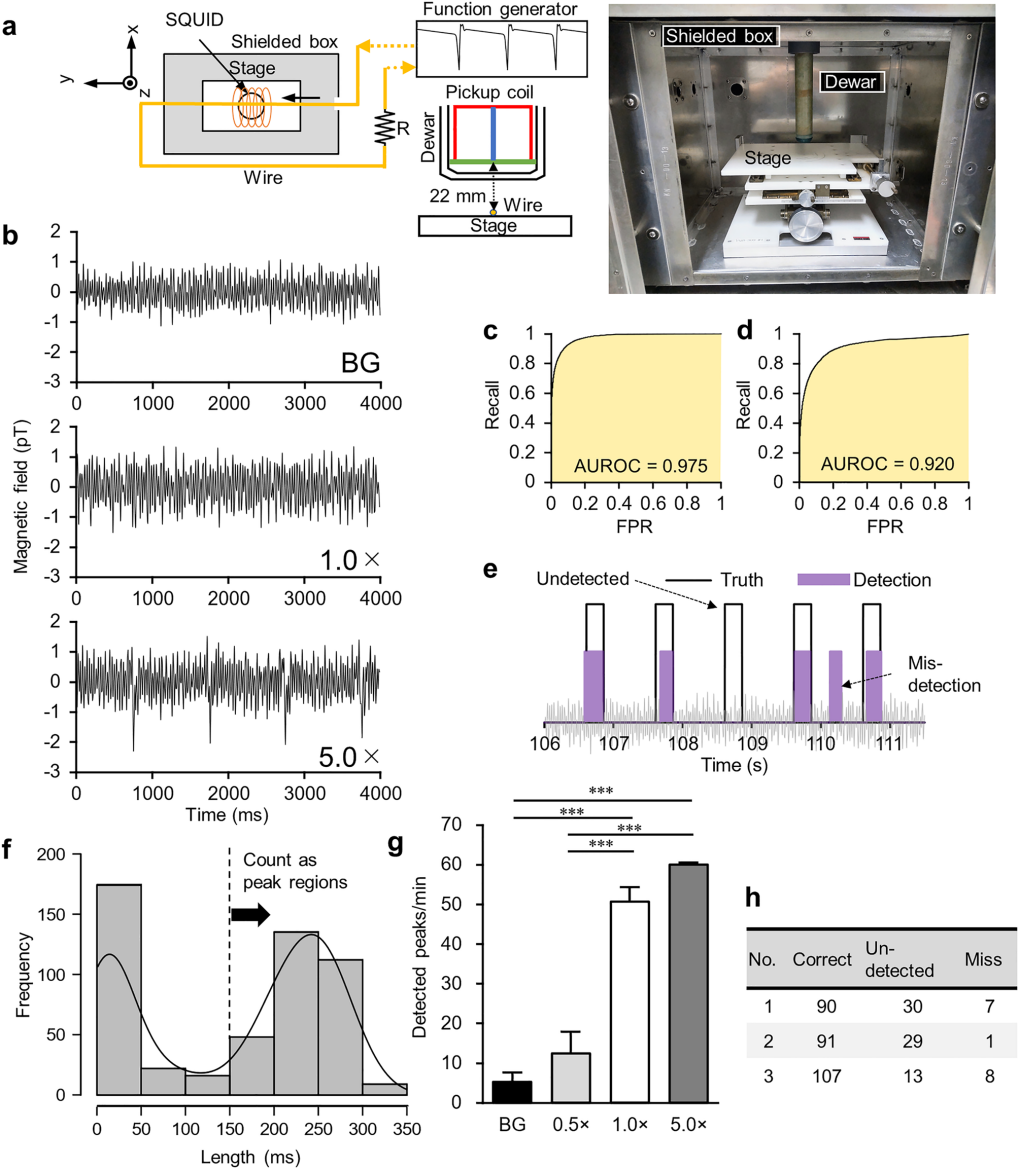
Validation of the proposed method using artificially generated magnetic signals. (**a**) Schematic diagram of the positional relationship between the wire generating a magnetic signal and the SQUID magnetometer. The photo on the right shows the inside of the MSB. (**b**) Representative measurement data are shown. From top to bottom, this is the measured data when no current was applied (background data), an estimated magnetic signal was generated, and a magnetic signal five times stronger than the estimate was generated. (**c**) ROC curve for the test data. The AUROC was 0.975. (**d**) ROC curve for the classification results against the experimental data. The AUROC was 0.920. (**e**) Peak regions detected from the 1.0 × data and the actual peak positions are superimposed. The solid gray line indicates raw magnetic waveforms. (**f**) Histogram of segment lengths labeled as P class. Results from three 1.0 × data were aggregated. The solid black line indicates density estimation. (**g**) Number of peaks detected from data with different signal intensities. Data are shown as mean ± SE (*n* = 3 for each intensity). *** *p* < 0.001. (**h**) Breakdown of the detection results from three 1.0 × data during 120 s. The number of correct matches to the true peaks, undetected peaks, and mis-detections, respectively.

We experimented with artificially generated magnetic signals to verify the feasibility of the proposed method. We generated magnetic signals that mimic magnetic fields from mouse iPS-CMs using a function generator that could output an arbitrary waveform voltage. Because we recorded the peak occurrence time simultaneously, we could use artificially generated magnetic signals as the ground truth to evaluate the peak detection capability of deep learning. We generated a magnetic field in the shielded box by passing an electric current through a wire under the sensor. To carry a linear current over a sufficiently long distance, we placed the wire along the *y*-axis and recorded the *x* component of the magnetic field (Fig. 4a). The generated magnetic field was approximated as

3 $$\begin{array}{*{20}c} {B = \frac{{\mu_{0} I}}{2\pi r}} \\ \end{array}$$

where *I* is the amplitude of the current and *r* is the distance from the wire to the measurement positions. We estimated the observed magnetic field by integrating Eq. (3) over the area of the pickup coil. We applied a current that generated a magnetic field that repeated at 1 Hz the peak waveform cut out from the simulation data. Representative measurement data from the SQUID magnetometer are shown in Fig. 4b. From top to bottom, the results show the measured data when no current was applied (background data), an estimated magnetic signal was generated, and a magnetic signal five times stronger than the estimate was generated. Note that the higher noise level than that during the cell sample experiment was caused by the *x*-direction measurement and the wire's presence. Therefore, we adjusted the intensity of the artificial signal so that the signal-to-noise ratio was the same as that in the cell sample experiment. We took three 120 s measurements for each intensity. Hereafter, we use the intensity multiplier to the estimated magnetic signal as a notation for the data. We used 5.0 × data, in which peaks could be discerned with the naked eye, as a positive control.

After network training, we computed the area under the receiver operating characteristic curve (AUROC)^36^ for the test data to evaluate classification performance (Fig. 4c). The AUROC was 0.975, which indicated high classification performance. Next, we used the trained network to classify the experimental data. The AUROC calculated with ground-truth data was 0.920, which was lower than that for the test data, but we still considered this network to have good classification performance (Fig. 4d).

Based on a histogram of the segment lengths labeled class P (Fig. 4f), we determined segments longer than 150 ms as peak regions. An example of superimposing the peak regions detected from the 1.0 × data and the actual peak positions is shown in Fig. 4e. Figure 4g shows the comparison of detection for different signal intensities. The number of detected peaks increased in proportion to the signal intensity, and we detected significantly more peaks in the 1.0 × data (*p* < 0.001; 50.7 ± 3.5 peaks/min) than in the background data (5.3 ± 2.1 peaks/min). A breakdown of the results from the three 1.0 × data during 120 s is shown in Fig. 4h. We detected 80.0% (= 288/360) of the peaks correctly. Additionally, we detected only 16.7% of the peaks in the 0.5 × data and detected all the peaks in the 5.0 × data (Supplementary Table S2).

### Effect of the frequency range and noise data on peak detection, and comparison with the scaled template technique

**Figure 5:**
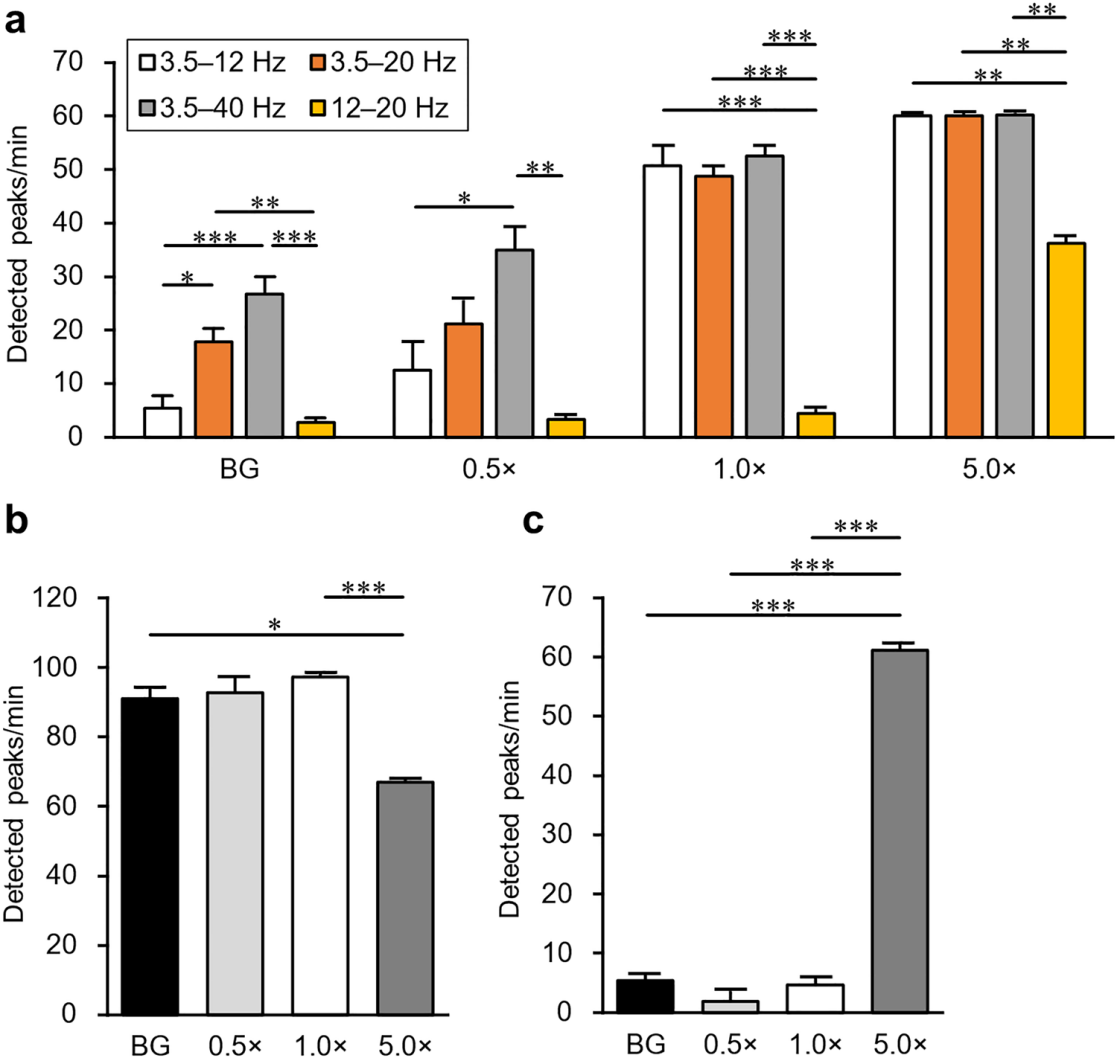
Effect of the frequency range and noise data on peak detection and result from the scaled template technique. (**a**) Comparison of the number of peaks detected by networks trained with the FSST frequency ranges of 3.5–12 Hz, 3.5–20 Hz, 3.5–40 Hz, and 12–20 Hz, respectively. The horizontal axis is the intensity of the artificially generated magnetic signals. (**b**) Number of peaks detected by the network trained using only simulation data without superimposing background noise data. (**c**) Number of peaks detected by the scaled template technique. All data are shown as mean ± SEM (*n* = 3 for each intensity). *p < 0.05; **p < 0.01; ***p < 0.001.

In the analysis described in the previous section, we used the 3.5–12 Hz frequency range of the spectrum calculated by the FSST as the data input into the network. To confirm the effect of the frequency range on peak detection, we additionally trained LSTM networks in five patterns and compared the detection results: 3.5–20 Hz, 3.5–40 Hz, 12–20 Hz, 20–40 Hz, and raw data without the FSST (Fig. 5a). Using the network trained in the 20–40 Hz range and the network trained on raw data, we detected no peaks from the data of any signal intensity. Therefore, these results are not shown in the plot. False positives from the background data significantly increased when the usage range was 3.5–20 Hz and 3.5–40 Hz (*p* = 0.019; 17.8 ± 2.2 peaks/min and *p* < 0.001; 26.7 ± 3.3 peaks/min) compared with 3.5–12 Hz. From the 1.0 × data, the number of detected peaks was significantly lower in the 12–20 Hz frequency range than the others (*p* < 0.001; 4.3 ± 0.9 peaks/min). For the 5.0 × data, we detected almost all peaks, but only 60.0% of the peaks when the usage range was 12–20 Hz. The details of the results are shown in Supplementary Table S2. We considered these results affected by the large background noise component around 12–20 Hz (Supplementary Fig. S4). We were satisfied with the low false positives from the background and decided to continue using the 3.5–12 Hz frequency range to train the network.

We also assessed the detection accuracy when we trained the network using only simulation data without superimposing background noise data. The detected peaks unexpectedly increased for data other than 5.0 ×, and approximately half were false positives (Fig. 5b and Supplementary Table S2).

Additionally, to compare deep learning with another peak detection method, we tested the scaled template technique^22^, which is a pattern matching-based method that is effective in the detection of small synaptic events, on the same measured data. As a result, we could not detect most peaks except for the 5.0 × data (Fig. 5c).

### Detection of magnetic signals from iPS-CMs

**Figure 6:**
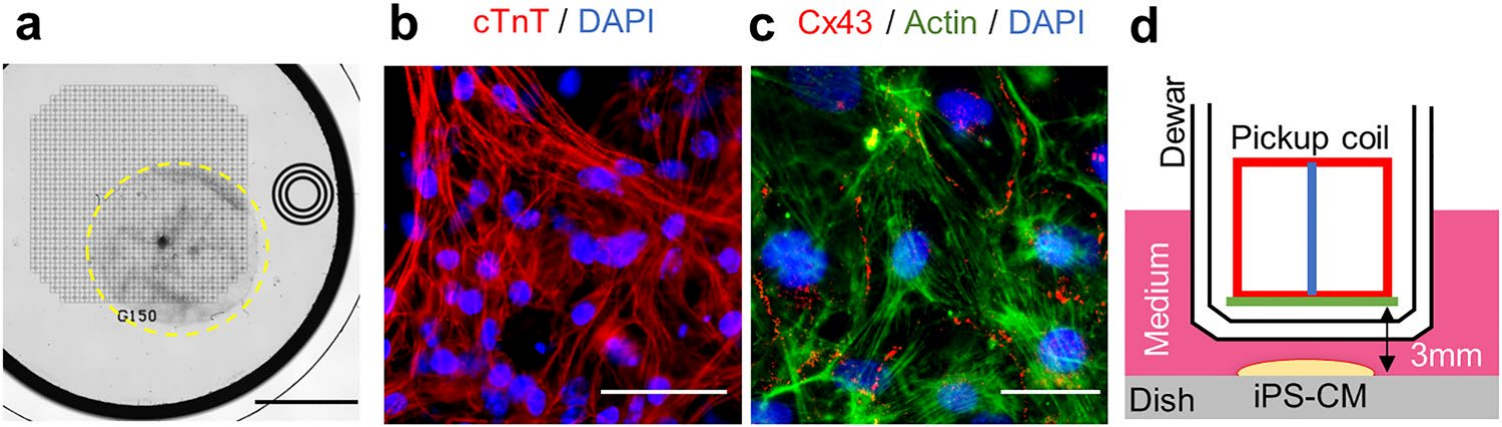
Preparation of mouse iPS-CM samples. (**a**) Mouse iPS-CMs adherently cultured on a grid-printed glass bottom dish. Cells are spread within the yellow dashed circle, scale bar = 3 mm. (**b**) Mouse iPS-CMs on day 19 of differentiation were immunostained with anti-cTnT (red), scale bar = 40 µm. (**c**) Stained with anti-connexin 43 (red), scale bar = 20 µm. In both (**b**) and (**c**), nuclei were stained with DAPI (blue). In (**c**), actin was stained with phalloidin (green). (**d**) Positional relationship between the SQUID magnetometer and the cell sample on the 100 mm plastic dish.

Experiments with artificially generated magnetic signals suggested the feasibility of the proposed method. Therefore, next, we attempted to detect magnetic signals from actual cells. We generated embryoid bodies (EB) from mouse iPS cells and induced them to differentiate into cardiomyocytes using adherent culture^37^. On days 19–21 of differentiation, we selected cell samples in which only one single synchronized beating cluster spread over a large area (> 3 mm square) was observed for magnetic measurement. An example of bright-field images of iPS-CMs cultured on a grid-printed glass bottom dish is shown in Fig. 6a. Immunostaining of the beating cluster revealed the expression of cardiac marker troponin T (Fig. 6b). We also observed the presence of connexin 43 in the intercellular region (Fig. 6c), which confirmed the formation of electrical connections via gap junctions. We used cells cultured in 100 mm plastic dishes or multi-electrode arrays (MEA)^38^ for magnetic measurement. Considering the influence of the material of the culture dish on magnetic fields, we analyzed the number of detected peaks separately for each type of dish. The positional relationship between the sensor and the sample during measurement is shown in Fig. 6d. We considered the starting point of the excitation propagation estimated using microscopic observation to be the position of the cluster of pacemaker cells. We placed the sample in a manner consistent with the conditions of the numerical simulation.

**Figure 7:**
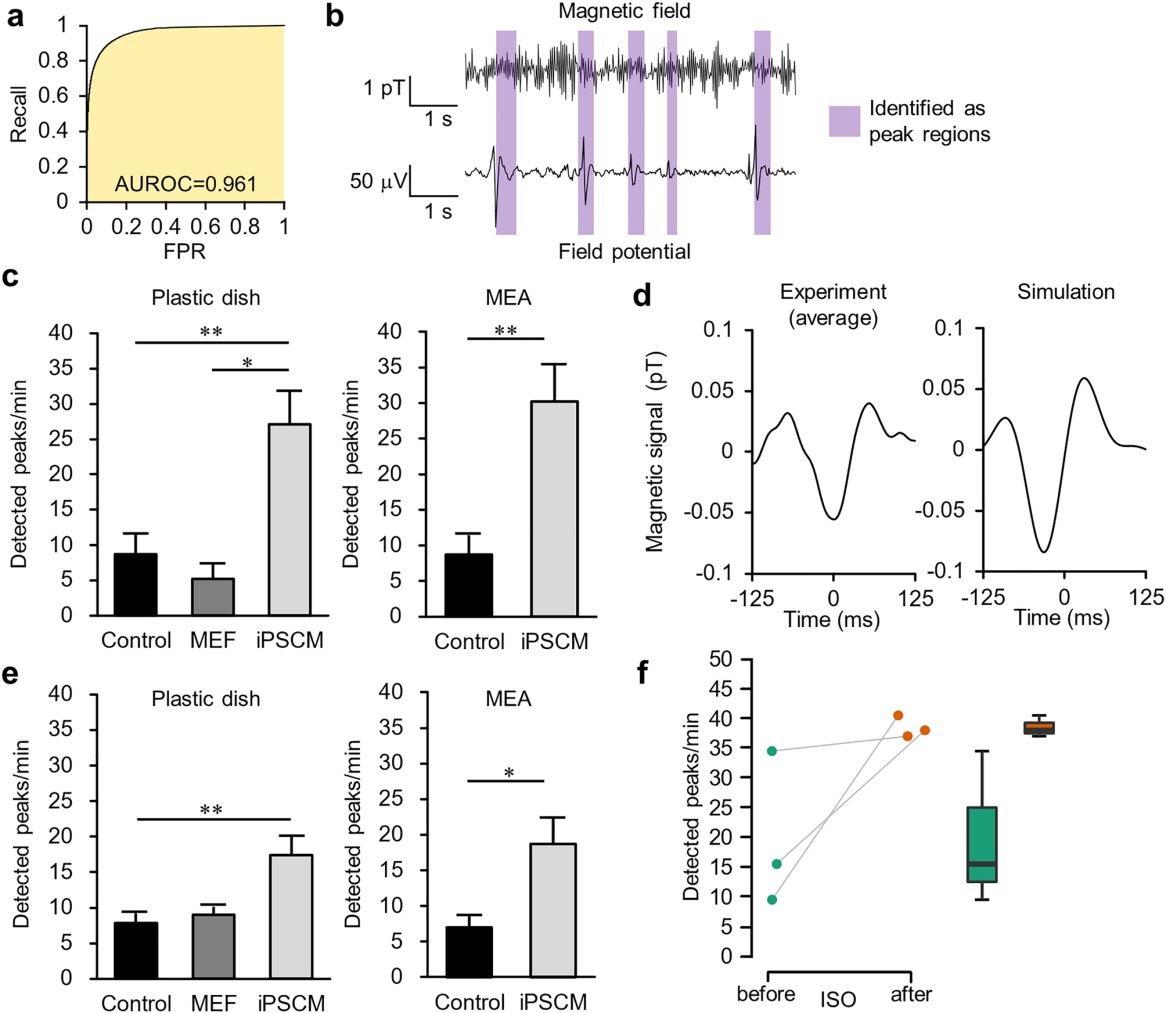
Detection of magnetic signals from iPS-CMs. (**a**) ROC curve for the test data. The AUROC was 0.961. (**b**) Waveforms of the magnetic field and the FP. We measured them simultaneously on MEA. The peak regions identified from the magnetic field by the network are indicated in purple. (**c**) Number of peaks detected from mouse iPS-CMs. We detected a significantly higher number from iPS-CMs on plastic dishes (*n* = 9) than the control group (*n* = 10) or MEFs (*n* = 3). Similarly, iPS-CMs on MEA (*n* = 8) showed a significant difference compared with the control group (*n* = 8). We obtained the data for the MEA samples used in this comparison when only magnetic measurements were performed. (**d**) The left panel shows the waveform averaged over all regions identified as peaks from one cell sample. The right panel shows the waveform estimated using numerical simulation. We applied LPF at 12 Hz to both waveforms. (**e**) Number of peaks detected by the network trained by the dataset with a fixed cycle length and amplitude. (**f**) Comparison of the number of peaks detected from data measured before and after we treated iPS-CMs (*n* = 3) with isoproterenol (final concentration: 10 μM). A boxplot is displayed on the right. We made this comparison using the paired *t*-test. All data are shown as mean ± SEM. *p < 0.05; **p < 0.01.

Using the network trained for cell experiments, we calculated the AUROC to be 0.961 for the test data (Fig. 7a). The network classified each data point, and we determined the peak regions based on its output. Like experiments for artificial signals, we set the minimum length for determining peak regions to 150 ms. The waveforms of the magnetic field and the FP measured simultaneously on MEA are shown side by side in Fig. 7b. The peak regions detected from the magnetic field are visualized as colored bands. We observed synchronization in the peak positions of both signals. Figure 7c shows the comparison of the number of detected peaks from mouse iPS-CMs with that from the control group. The control was a cell-free sample containing medium only in a plastic dish or MEA. We also compared iPS-CMs on plastic dishes with mouse fetal fibroblast (MEF) samples, which are not excitable cells. The numbers of detected peaks from iPS-CMs were significantly higher (*p* = 0.004; 27.1 ± 4.6 peaks/min for plastic dishes and *p* = 0.003; 30.2 ± 5.3 peaks/min for MEA) than those from the control group (8.7 ± 2.7 peaks/min for plastic dishes and 8.7 ± 2.8 peaks/min for MEA) and MEFs (5.2 ± 2.0 peaks/min). The left panel of Fig. 7d shows the waveform averaged over all regions identified as peaks from one iPS-CMs sample on a plastic dish. The right panel shows the peak waveforms estimated by the simulation, and we observed their similarity.

Figure 7e shows the detection results when we trained the network using magnetic signals with a fixed cycle length and amplitude (i.e., no variability). The number of peaks declined from that detected by the default network. After subtracting the number of peaks detected from the control group, we compared the result to beats per minute (BPM) counted by microscopic observation before magnetic measurement (Table 1). The number of peaks detected by the default network was in good agreement with the BPM. By contrast, when we trained the network using data without variability, the number of detected peaks was significantly lower than the BPM (*p* = 0.036 for plastic dishes and *p* = 0.015 for MEA). These findings suggest that the detected magnetic signals reflect the actual electrical activity of the cell population and that the addition of experiment-based variability to the training data contributes to the classification ability of the network.

**Table 1:** Comparison of the number of peaks detected using deep learning and BPM counted using microscopic observation. Plastic dish: *n* = 6 and MEA: *n* = 5. All data are shown as mean ± SEM. The detected peaks from the control group were considered false positives and their mean number was pre-subtracted.

Dish type	Training data	Detected peaks/min (subtracting control)	BPM
Plastic dish	Default	19.5 ± 6.8	20.6 ± 2.3
	No variability	12.2 ± 3.3	
MEA	Default	18.1 ± 3.2	24.6 ± 3.7
	No variability	8.4 ± 1.2	

Finally, we performed magnetic measurement before and 30 min after the administration of 10 μM isoproterenol to iPS-CMs samples on plastic dishes to verify whether we could detect the chronotropic effect of the drug^39^. As a result, there was a peak increase trend from 19.8 ± 7.5 peaks/min to 38.5 ± 1.0 peaks/min before and after administration (Fig. 7f).

## Discussion

In this study, we demonstrated the effectiveness of deep learning in the detection of weak magnetic signals from iPS-CMs. We solved the problem of the difficulty of obtaining labeled training data using simulated data as an alternative. We identified peak regions of biomagnetic signals using classification with LSTM networks and evaluated the rhythm and changes in cellular electrical activity. Compared with adult cardiomyocytes, iPS-CMs are less mature and their conduction velocity is much slower^40^. Therefore, the generated magnetic signal was also weaker than anticipated and, as we showed in our simulations, two or three orders of magnitude smaller than the results previously reported from the magnetic measurements of other cells^10,11^. Its intensity was close to the background noise, and it was not easy to identify the peak using techniques such as visual inspection or noise filtering. For example, in the waveform of the measured data after denoising with LPF, the region of the peaks was still unclear (Supplementary Fig. S5). One of the advantages of deep learning is that it requires no prior knowledge or manual preprocessing of the target, and the features used for classification are extracted automatically^9^. It is considered to be suitable for the analysis of magnetic signals from iPS-CMs, for which the information of waveforms is still insufficient.

We obtained the best results in experiments with artificially generated signals when the frequency range was limited to 3.5–12 Hz. This result is presumably caused by environmental factors, such as noise centered on 12–20 Hz caused by elevators and air conditioning in the building^41^, rather than signal characteristics. False positives significantly increased when we performed classification using the network trained only using simulation data without superimposing background noise. This result suggests that learning not only the signal but also the characteristics of noise is essential for the classification task. Therefore, by increasing the variety of noise data, it may be possible to reduce the number of false positives, which are currently not negligible. From these factors, it may be necessary to update noise data and tune the frequency range each time the measurement environment or equipment changes. Since differences in noise between laboratories (for example, the frequencies of the commercial power supply that generates hum noise are different, 60 Hz or 50 Hz, depending on the country) cannot be ignored, we would like to confirm the robustness and reproducibility of the proposed method in multiple locations. By contrast, once we completed network training, the proposed method was superior in throughput because the time required for the classification task on the measured data was much shorter than for training. In addition, an optically pumped magnetometer, which has sensitivity approaching SQUID and does not require cooling, thus reducing running costs, has attracted much attention recently^42^. Combining this novel miniaturizable sensor with deep-learning data analysis may increase the feasibility of high-throughput, large-scale drug screening. Another cutting-edge technology is bioprinting, which can automate the plating process of iPSC-CMs without compromising drug sensitivity^7^. Replacing manual culture with a bioprinter could help speed up and automate the proposed method. We also demonstrated the feasibility of weak biomagnetic signal detection in a general laboratory environment without special and expensive magnetic shielding, such as building-level shielding.

Measuring cells that form an electrically coupled functional syncytium with a single beating region is essential for one-to-one correspondence between the magnetic signal and the electrical activity of cells^43^, but it is not easy to select such cells strictly. In a future task, we will verify the influence of multiple beating regions on the observed signals using both experiments and numerical simulation.

We estimated the amplitude of the magnetic field based on the assumption of isotropic current flow in the cells. However, actual cells have considerable anisotropy and distribution bias, and the magnetic field is expected to be asymmetric. In this study, we used only the y-axis direction magnetic field, which has relatively low noise, for analysis, but it will be needed to verify the directional dependence in a less noisy environment.

Because the spontaneous beating rate of mouse iPS-CMs does not differ significantly from the heart rate of adult humans^24^, we used mouse iPS-CMs^44^ in this study as a preliminary experiment. Despite this, it is preferable to use human iPS-CMs^45^ when considering applications such as drug safety tests and the evaluation of regenerative medical materials. However, the conduction velocity of human iPS-CMs may be slower than that of mice^40^, and their magnetic signals are predicted to be weaker. Therefore, magnetic measurement for scaled-up samples, such as myocardial sheets cultured at high density^46^, will be necessary. Furthermore, the Mercola Laboratory has recently developed maturation media that suit the metabolic needs of human iPS-CMs^47^. The media improve the physiological functions of human iPS-CMs compared to existing media and increase the fidelity of modeling cardiac disorders. In particular, the expression of the channel that conducts the K^+^ current I_K1_, which was weak in conventional iPS-CM, is enhanced, so the magnetic signals from samples cultured in this medium are expected to be close to those of adult human cardiomyocytes. Beat rate and AP shape differences have been observed at various stages of cardiomyocyte maturation^24^. If magnetic measurements can detect these changes, they could be applied to non-invasive monitoring of cell maturation in regenerative medicine. In addition, it has been reported that responses to drug administration are not the same among cell lines established by different methods^29^. Although a single cell line was used in the present study, comparing the degree of variation in magnetic signals between the different types of samples will be interesting.

It is also desirable to perform the measurement at 37 °C, the same as the biological environment, instead of at room temperature. The problem is that standard temperature maintenance equipment generates electrical noise, which makes it difficult to achieve compatibility with magnetic measurements. Therefore, we are considering introducing devices that do not affect the magnetic field, such as an isothermal pad based on a thermodynamic principle.

The peak number of the magnetic signal estimated using numerical simulation was 57.1 peaks/min, but the beating rate of the actual cells was less than half of that (Table 1). The reason for this discrepancy may be that the AP model of pacemaker-like cells was optimized based on a classical and simplified model compared with ventricular-type cardiomyocytes, and therefore could not adequately reproduce the rhythm of electrical activity. By contrast, the fact that the estimation of the peak waveform was correct was supported by good agreement with the waveform obtained by averaging the detection regions. We speculate that this difference may be because the contribution of the current between ventricular-type cardiomyocytes to the magnetic waveform is more remarkable than the current between pacemaker-like cells. In any case, when changing the measurement target to human iPS-CMs, optimization of a new AP model and reconstruction of the biomagnetic simulation will be mandatory.

Providing variability in the training data improved detection performance. To ensure diversity closer to real-world data, we plan to introduce cell-level variation^48^. The number of detected peaks after the administration of isoproterenol showed a trend toward an increase from the pre-dose level. By contrast, not only the period but also the waveform may have changed after drug administration^39^. Improving the training data using in silico experiments to estimate the magnetic waveform during drug administration may enable more accurate detection. A previous study^20^ that reported a deep learning network trained with simulated action potential data to classify cells into drug-free and drug-treated categories is highly suggestive. Moreover, we classified the measurement data into two classes, but it is possible to subdivide the classification into more categories, like the P wave, QRS, and T wave in ECG. This extension would be beneficial in creating indicators of abnormal electrical activity, such as QT prolongation in ECG and FP duration prolongation in FP^49^.

FP measurement is an excellent method for detecting the electrical activity of cardiomyocytes because the electrodes are in contact with the cell and provide a stable signal with little noise^38^. In a previous study^50^, FPs were numerically calculated using AP models of iPS-CMs, and the effect of drugs on electrical activity was compared with experimental data. FP measurement is currently the mainstream approach for drug screening applications, but the difficulty of recovering cells after measurement is a drawback as a detection device in regenerative medicine for human transplantation. Membrane potential-sensitive fluorescent dye has similar problems because it is invasive. Magnetic measurement, which is non-invasive and not restricted to specific culture devices, has advantages in this respect. However, this method is still inferior to other methods regarding peak detection accuracy and reliability of averaged waveforms. One approach to improving these problems is to adjust the width of the Kaiser window used in FSST, which converts magnetic signals into frequency domain data. There is a trade-off relationship where making the window wider increases the resolution in the frequency domain, and making it narrower increases the resolution in the time domain. The results of learning the network with different widths and detecting peaks from the artificial signal data are shown in Supplementary Fig. S6. When a width of 1024 was used, false positives from the background significantly increased compared to the others. When a width of 256 was adopted, there was no significant difference from using the default width of 512, but a slight improvement was observed in the number of peaks detected from 1.0 × data. Therefore, this method could be refined by increasing the time domain resolution.

Magnetic signals from cells reflect the anisotropy of electrical propagation and the state of the cell distribution^11,51,52^, and signal transmission is not attenuated by living tissue^53,54^. Thus, in the future, the method demonstrated in this study may provide electrophysiological information previously undetectable from membrane potentials and FPs.

## Methods

### Genetic algorithm

We used the GA to optimize the conductance of each current so that the AP model reproduced the experimental values in previous studies^24,29^. We executed the GA optimization using a program implemented in C# with reference to the method of Bot et al*.*^55^, and its type was a real-coded GA. We evaluated the degree of adaptation of each model in the population using the score calculated using Eq. (1). We calculated the AP for each model 60 s after the initial state. We performed numerical integration to compute APs using the forward Euler method with a time step of 0.01 ms. The initial values of ion concentration inside and outside the cell, and temperature were equivalent to the conditions of the experiments. We fixed the intracellular potassium and sarcoplasmic reticulum calcium concentrations to accelerate convergence. We estimated the cell volume from the cell surface area data^24,56^. We used the same value as that in the Paci model for the ratio of the sarcoplasmic reticulum volume to the cytoplasmic volume^23^. We used a model population with random values assigned to each conductance as the starting generation. The upper and lower scaling limits were 0.0–10.0 for *G*_Na_, *G*_CaL_, and *G*_f_ in the ventricular-type model and 0.0–5.0 for all others. We describe the details of the GA optimization of AP models in the Supplementary Methods.

### 2D simulation

To estimate magnetic signals from iPS-CMs, we simulated the 2D electrical activity of the cell population. We set the intracellular ion concentrations using adult mouse cardiac AP model values^57^. We determined the extracellular ion concentrations from the composition of the culture medium and set the temperature to room temperature (RT: 24 °C). The temperature coefficients (Q_10_) used in the AP models referred to values from the published literature^58^. We computed the solution to the partial differential Eq. (2) using the Crank–Nicolson method, with the spatial step set to Δ*x* = Δ*y* = 60 μm and the time step set to Δ*t* = 0.01 ms. We determined the averaged cellular resistivity to reproduce the conduction velocity measured in neonatal rat cardiac cell sheets^40^ (Supplementary Methods and Supplementary Fig. S7). The list of parameters used in the simulation is summarized in Supplementary Table S3. We calculated the magnetic field using Biot–Savart's law from the currents flowing in the cells at each time point. We estimated the observed waveforms using integration in the area of each pickup coil. We assumed that the cancellation component of the magnetic field caused by the extracellular return current was negligibly small because the volume of the medium was sufficiently large relative to the spreading of cultured cells. We also checked how much the observed waveforms were affected when the cell position was shifted from directly under the sensor. As a result, we confirmed that a displacement of ± 2 mm in the *x*-axis or *y*-axis directions had almost no effect on the measurements (Supplementary Fig. S8).

### Training dataset

The procedure for dataset preparation is as follows: A peak region (250 ms) was cut from the magnetic signals estimated using simulation and subjected to random stretching and scaling. Non-peak regions between peak regions were linearly interpolated to make data of 120 s each. For the background noise, four data of the *x* component of the magnetic field with no current applied (for artificial signal experiments) or eight data of the *y* component with no cell sample placed (for cell experiments) were used in equal proportions within each dataset. Random time shifts were performed in the superposition of these magnetic signals and noise data. Even when the cycle length and amplitude were fixed, this shift brought diversity to the dataset. Finally, datasets (*n* = 160 for artificial signal experiments or 640 for cell experiments) were generated, including three data types in a 1:1:2 ratio: positive peak direction, negative peak direction, and background noise only. Representative waveforms are shown in Supplementary Fig. S9.

### Configuration of the network training parameters

The window used in the spectral calculation with the FSST^33^ was the Kaiser window, with a size of 512 points. The sidelobe attenuation was 13.6 dB. The real and imaginary parts of the spectral were input as separate features. The input values were pre-standardized by subtracting the mean and dividing by the standard deviation. Training was iterated for up to 60 epochs (one epoch means one round of data). The network was validated using the validation data for each epoch. If the validation loss exceeded the previous minimum value more than ten times, it was decided that there was no further improvement and training was stopped. The initial learning rate was set to 0.001 and the learning rate was dropped by a factor of 0.1 every 20 epochs. The training data were divided into segments of 10 s lengths and the mini-batch size (a subset of the training data used in one step to evaluate the gradient of the loss function and update the weights) was set to 16.

### Evaluation of network classification performance and determination of peak regions

We calculated the AUROC^36^ to evaluate network classification performance. The AUROC is the area under the curve plotted with the false positive rate on the horizontal axis and the true positive rate on the vertical axis. The AUROC is 1.0 when separation performance is best and 0.5 when classification is performed randomly. In this study, we defined each data point as positive if it was peak (P) or negative if it was non-peak (N).

From the output label data, we plotted a histogram of the lengths of segments labeled as class P (Fig. 4f). Using this histogram as a reference, we estimated the appropriate distribution of peak region lengths, set a lower limit, and identified segments longer than this threshold as peak regions (Fig. 1c). We used the average count number for the analysis of samples measured multiple times. We obtained the average waveform by superimposing magnetic signals of 175 ms before and after the center position of each peak region and averaging their amplitudes. Then, we repeated the adaptive correlation filter^59^ ten times to correct for positional fluctuations.

### Magnetic measurement instruments

A vector-type SQUID magnetometer^12,15^ was applied to measure magnetic fields. The vector-type SQUID magnetometer had an axial-type first-order gradiometric pickup coil with a diameter of 15.5 mm and two planar-type first-order gradiometric squared pickup coils of 9 × 15.5 mm^2^ and 11 × 15.5 mm^2^. The baseline length of each gradiometric pickup coil was 50 mm. The three gradiometric pickup coils were oriented perpendicular to each other and assembled on a cylindrical bobbin. Three Ketchen-type low-temperature SQUIDs were individually coupled to each pickup coil and simultaneously detected the three independent components of the magnetic field: B_x_, B_y_, and B_z_. The SQUID readouts were connected to double-integrator type flux-locked loop (FLL) circuits for output linearization and dynamic range improvement. The total noise level, including environmental noise, was 10–20 fT/√Hz at 10 Hz. The SQUID magnetometer was installed in a glass-fiber reinforced plastic (GFRP) cryostat with an MSB. The MSB comprised two 1 mm thick mu-metal layers with double front doors. The shielding factor of the MSB was more than 40 dB at 10 Hz. The GFRP cryostat consisted of a cylindrical main body that stored 6-L liquid helium and a narrow GFRP tube that dropped from the bottom of the main body. The main body was installed in the ceiling of the MSB and only the GFRP tube penetrated the MSB through a hole in its top. The SQUID magnetometer was installed at the bottom end of the GFRP tube and placed at the center of the MSB.

The cell sample was placed on a height-adjustable stage made of non-magnetic materials and adjusted to 3 mm from the bottom edge of the pickup coil. Measurements were taken at room temperature, and the FLL readout signals were digitally recorded at the sampling rate of 1 kHz with HPF at 3 Hz, LPF at 100 Hz, and notch filters at 60 Hz.

### Adjustment of artificially generated magnetic signals

We kept the resistance fixed and varied the output voltage of the function generator to adjust the current that generated magnetic signals. With no filtering, we increased the voltage until the peak of the magnetic signal could be identified by visual inspection and recorded the peak amplitude at that point. Based on that value, we adjusted the voltage to generate the desired magnetic signals. We enhanced the artificial signal (2.74 ×) so that the signal-to-noise ratio was equivalent to that in the cell sample experiment. To confirm the validity of this procedure, we compared the amplitude spectrum densities of the background noise between the artificial signal experiment and the cell sample experiment (Supplementary Fig. S4). Although differences in amplitude existed, the spectral distribution had the same trend within the range of 3.5–40 Hz used to train the LSTM networks.

### Scaled template technique

We implemented the scaled template technique following previous research^22^. We slid the template (the event waveform of interest to detect) along the time series data and scaled it to fit the data at each position. Then, we divided the template scaling factor by the standard error of the time series data, which was the detection criterion, and we considered the event waveform of interest to be detected when this criterion exceeded a threshold value. The template was a peak waveform of 250 ms in length cut from the magnetic signal estimated using numerical simulation, which we also used as the training data in deep learning. To compare the two methods without bias, we set the threshold so that the number of detected peaks from background noise was equal to that of deep learning.

### Preparation of differentiated cardiomyocytes

The mouse iPS cell line iPS-MEF-Ng-20D-17 (Expressing GFP by Nanog promoter)^44^, established by the Center for iPS Cell Research and Application, Kyoto University, was provided by the RIKEN BRC through the National BioResource Project of the Ministry of Education, Culture, Sports, Science, and Technology, Japan. For the culture method, we referred to previous studies^44,60,61^. To maintain the undifferentiated state of iPS cells, MEFs (EmbryoMax® Primary Mouse Embryonic Fibroblasts, PMEF-NL, Neo Resistant, Strain FVB; purchased from Sigma-Aldrich, St Louis, MO, USA), in which cell proliferation was arrested by mitomycin C (Nacalai Tesque, Kyoto, Japan) treatment, were cocultured as feeder cells. The maintenance medium was composed of Dulbecco's modified Eagle's medium (Sigma-Aldrich) with 15% fetal bovine serum (Equitech-Bio Inc., Kerrville, TX, USA), 50 U/ml penicillin, 50 µg/ml streptomycin (Sigma-Aldrich), 2 mM L-glutamine (Sigma-Aldrich), nonessential amino acids (100 ×) (Sigma-Aldrich), 0.1 mM 2-mercaptoethanol (FUJIFILM Wako Chemicals, Osaka, Japan), and 0.1% human leukemia inhibitory factor (FUJIFILM Wako Chemicals). The medium was refreshed daily and iPS cells were passaged every two days. Colonies were detached with 0.25% trypsin/1 mM EDTA (FUJIFILM Wako Chemicals), dispersed in cell suspension, counted, and 1.0 × 10^6^ cells were seeded into MEFs on 60 mm plates.

Based on previous studies^37,62^, cardiomyocyte differentiation was induced by forming EB. The differentiation medium was Iscove's modified Dulbecco's medium (Sigma-Aldrich) containing 20% fetal bovine serum, 50 U/ml penicillin, 50 μg/ml streptomycin, 2 mM L-glutamine, nonessential amino acids (100 ×), and 0.1 mM 2-mercaptoethanol. Mouse iPS cells were suspended at 1.5 × 10^4^ cells/ml in the differentiation medium and seeded 0.2 ml into each well of a 96-well U-shaped-bottom microplate (Nunclon Sphera; Thermo Fisher Scientific, Waltham, MA, USA). The plates had a cell-nonadherent surface treatment, which allowed uniform and stable EBs to form. For further differentiation, the culture was switched from floating to adherent on day 5. Plastic dishes of 100 mm diameter and MEA (Alpha MED Scientific, Osaka, Japan) were used for magnetic measurement, and glass bottom dishes (AGC Techno Glass, Shizuoka, Japan) were used for fluorescence microscopy. These dishes were coated with 0.1 w/v% gelatin solution (FUJIFILM Wako Chemicals) and one EB was transplanted at the center of each dish. Beating areas began to appear on day 7. Magnetic measurement was performed during days 19–21 when the area of differentiated cells was extensive and synchronized beating was observed. Fluorescence microscopy was also performed at this time. To ensure that one peak corresponded to the electrical activity of the entire cell population, samples with a single beating area larger than 3 mm square were selected for measurement. To bring the cells closer to the sensor, the cylinder of the MEA was excised to a height of 1 mm. For comparison with iPS-CMs, MEFs were also cultured in cloning rings with an inner diameter of 5 mm. In the experiment to detect the drug's chronotropic effects from magnetic signals, the medium was replaced with a medium supplemented with isoproterenol at a final concentration of 10 μM, and magnetic signals were measured from iPS-CMs after incubation for 30 min.

### Fluorescent staining

Cardiomyocytes were immunostained on day 19 of differentiation, and the expression of cardiomyocyte marker cardiac troponin T and connexin 43 that forms gap junctions was confirmed. iPS-CMs were fixed in 4% paraformaldehyde for 20 min at 4 °C, followed by blocking with 5% goat serum (Nichirei, Tokyo, Japan) and 0.1% Triton-X diluted in Dulbecco's phosphate buffered saline (DPBS) for 20 min at RT. The cells were washed three times for 5 min with DPBS and incubated with a primary antibody diluted in DPBS containing 1% goat serum for 1 h at RT and then overnight at 4 °C. The primary antibodies were rabbit polyclonal IgG anti cardiac troponin T antibody (1.4 μg/mL; Proteintech, Rosemont, IL, USA) and rabbit polyclonal IgG anti connexin 43 antibody (10 μg/mL; Thermo Fisher Scientific). The cells were washed three times for 5 min with DPBS with shaking and further incubated with the secondary antibody Alexa fluor 546 goat anti rabbit IgG (Invitrogen, Carlsbad, CA, USA; 1:1000 dilution in DPBS/0.05% Triton X-100) for 30 min at RT. The cells incubated with connexin 43 antibody were also treated with Alexa fluor 488 Phalloidin (Thermo Fisher Scientific; 1:50 dilution in DPBS/0.05% Triton X-100) and stained for actin filaments. The cells were washed three times with tris buffered saline for 5 min and once with DPBS for 5 min, and immersed in 4',6-diamidino-2-phenylindole (DAPI)-added anti-fading agent (Nacalai Tesque). Observation and imaging were performed with an IX71 fluorescence microscope (Olympus, Tokyo, Japan).

### Electrophysiology

We measured FPs using MEA^38^. We selected the electrode near the center of the beating area and recorded the potential difference between it and a reference electrode not in contact with the cells. To avoid noise when measuring simultaneously with magnetic signals, we output the electrical signals from the probe externally through an IC clip and did not use the attached connector. When measuring FP only, we used it. We performed the measurement at room temperature and recorded data at the sampling rate of 1 kHz with HPF at 0.16 Hz, LPF at 160 Hz, and notch filters at 60 Hz.

### Software

Deep learning network training and data classification were performed in MATLAB (Mathworks Inc., Natick, MA, USA). The GA for parameter optimization of the AP model and the numerical simulation of the electrical activity of cardiomyocytes were performed using our programs implemented in C#.

### Statistical analysis

Data are presented as mean ± standard error of the mean (SEM). Comparisons between two groups were analyzed using the unpaired *t*-test unless otherwise indicated. For comparisons of three or more groups, when equal variances could be assumed, one-way ANOVA was used, followed by Tukey's test as a post hoc test. When equal variances could not be accepted, the Brown–Forsythe correction was performed, followed by the Games–Howell test as a post hoc test. Differences between data were considered statistically significant at *p* < 0.05.

### Supplementary Information

Supplementary Information.

## Data Availability

The data used in this study are available from the corresponding author upon reasonable request.
