# Supplementary material for: Detection of biomagnetic signals from induced pluripotent stem cell-derived cardiomyocytes using deep learning with simulation data
Source: Sci Rep. 2024 Mar 27;14:7296. doi: 10.1038/s41598-024-58010-0 (PMC10973465; doi:10.1038/s41598-024-58010-0)
Supplement: Supplementary file 1 — Supplementary Information. [file 41598_2024_58010_MOESM1_ESM.pdf]

Detection of biomagnetic signals from induced pluripotent stem cell-derived cardiomyocytes using deep learning with simulation data

Takeshi Yamaguchi[1,2,*], Yoshiaki Adachi[2], Takashi Tanida[3], Katsutoshi Taguchi[1], Yoshinobu Oka[4], Takashi Yoshida[5], Wook-Cheol Kim[6], Kenji Takahashi[7], Masaki Tanaka[1]

[1]Department of Anatomy and Neurobiology, Graduate School of Medical Science, Kyoto Prefectural University of Medicine, Kyoto 602-8566, Japan
[2]Applied Electronics Laboratory, Kanazawa Institute of Technology, Ishikawa 920-1331, Japan
[3]Department of Veterinary Anatomy, Graduate School of Veterinary Science, Osaka Metropolitan University, Osaka 598-8531, Japan
[4]Department of Pediatric Orthopaedics, Graduate School of Medical Science, Kyoto Prefectural University of Medicine, Kyoto 602-8566, Japan
[5]Department of Orthopaedic Surgery, North Medical Center, Kyoto Prefectural University of Medicine, Kyoto 629-2261, Japan
[6]Department of Pediatric Orthopaedic Surgery and Ilizarov Center, Uji Takeda Hospital, Kyoto 611-0021, Japan
[7]Department of Orthopaedics, Graduate School of Medical Science, Kyoto Prefectural University of Medicine, Kyoto 602-8566, Japan

Supplementary Methods

Genetic algorithm

An overview flowchart of the genetic algorithm (GA) used in this study is shown in Supplementary Fig. S1. We implemented the GA optimization with reference to the previous study[1]. The parameters to be optimized were the scaling factors of each channel conductance. The population size was 240 (ventricular-type cardiomyocytes) or 120 (pacemaker-like cells), and the number of generations was 50.

First, a mating pool consisting of individuals with higher fitness is created using tournament selection from the population, and then random pairs are made. Next, two children are born from each parent pair, and a crossover occurs in which the parameters are swapped (with a probability of 0.9). The simulated binary crossover (SBX) technique[2] with polynomial order one and single parameter gene-wise swap probability of 0.5 is used for parameter replacement. If no crossover occurs, the two offspring inherit the parameters of the two parents. Then, there is the possibility of mutations that cause further changes in parameters. The probability of mutation is set to 0.125 for each conductance parameter. Parameter changes are calculated using a polynomial mutation operator[3] with order 20. Finally, as an elitism strategy, the best-fitting individual is left to the next generation (exchanged with the worst-fitting individual). This cycle is repeated, and the best individual is adopted as our optimized model at the end of 50 generations.

Conduction velocity and averaged cellular resistivity

Conduction velocity (CV) was calculated from a distance between two points along the diagonal direction from pacemaker-like cells to ventricular myocytes and the time difference of zero crossing points of action potential (AP). The CV of mouse induced pluripotent stem cell-derived cardiomyocytes (iPS-CMs) was assumed to be similar to the experimental value of 14.8 cm/s for neonatal rat myocardial sheets[4]. The averaged cellular resistivity $\rho = 60\ \Omega \cdot m$ was employed in the simulation, in which case the CV was estimated to be 14.1 cm/s (Supplementary Fig. S6).

Supplementary Figures

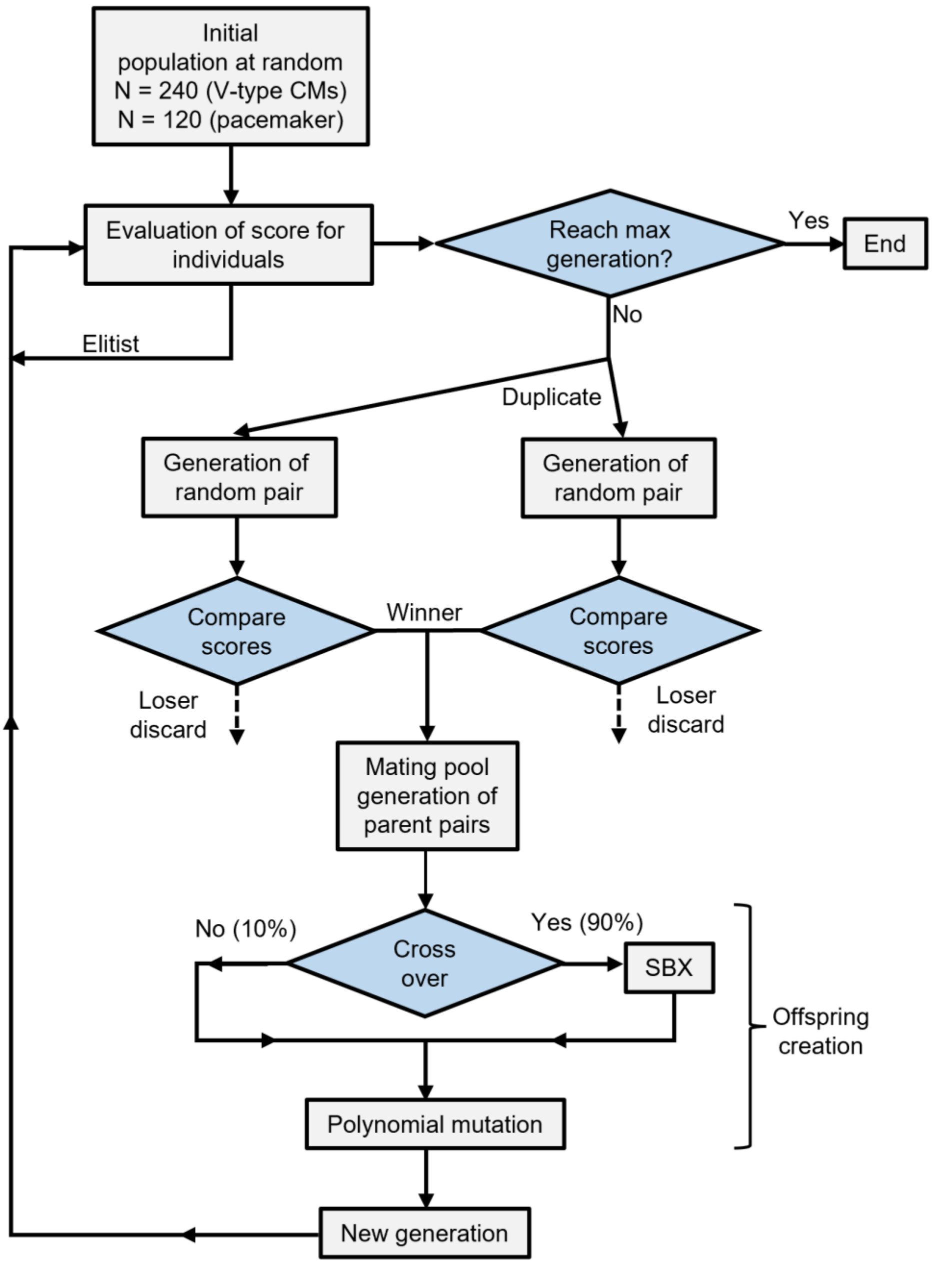

Supplementary Figure 1: Block diagram of the genetic algorithm.

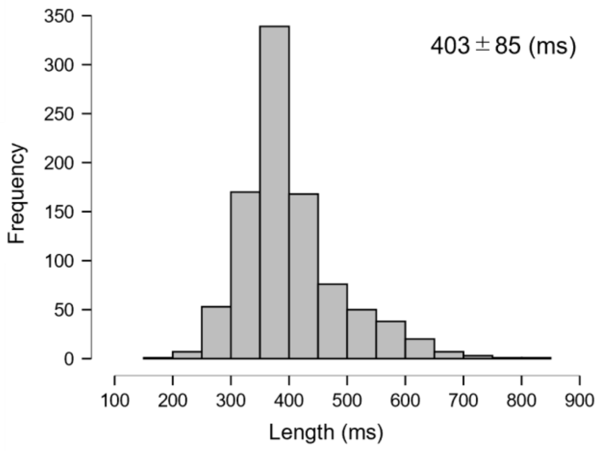

Supplementary Figure 2: Histogram of the peak region lengths of field potentials. We measured the field potentials of iPS-CMs on day 21 of differentiation with multi-electrode arrays and plotted the length distribution of peak regions. We identified peak regions manually. The total number of peak regions was 934. We estimated the coefficient of variation at 0.21.

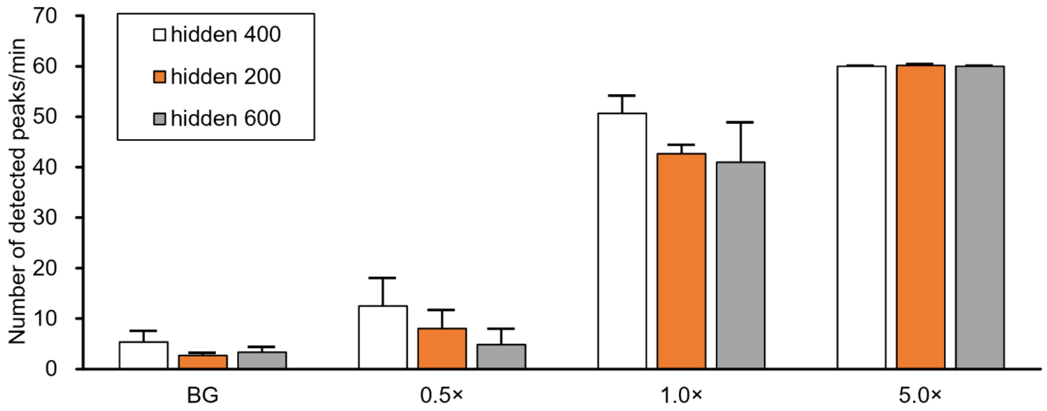

Supplementary Figure 3: Effect of the number of hidden units on peak region detection. We compared the number of detected peaks from artificially generated magnetic signals using networks trained with the number of hidden units in the long short-term memory (LSTM) layer set to 200, 400, and 600, respectively. The horizontal axis is the intensity of the artificially generated magnetic signals. We observed no significant change with the number of hidden units, but detection results from the 1.0× data were best when the number of units was 400.

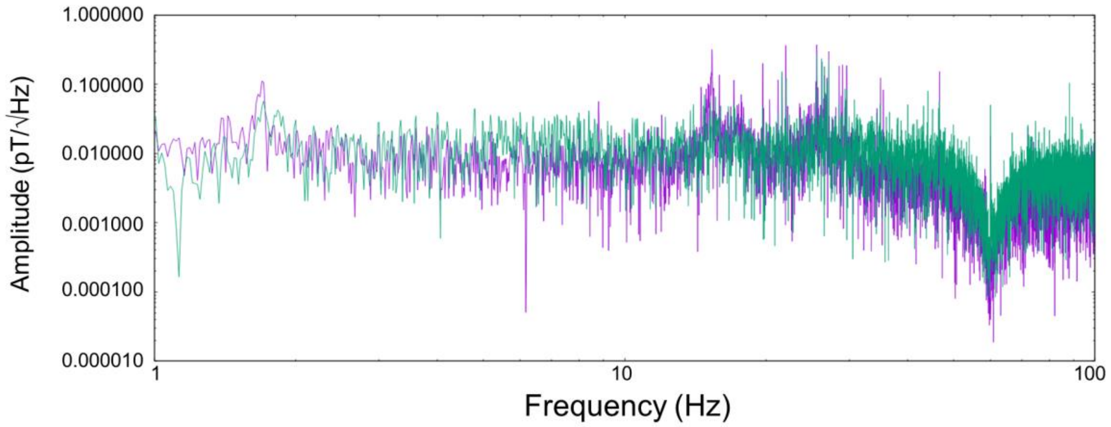

Supplementary Figure 4: Background noise comparison in the frequency domain. The Amplitude Spectrum Densities of background noise were compared between the artificial signal (green) and cell sample (purple) experiments.

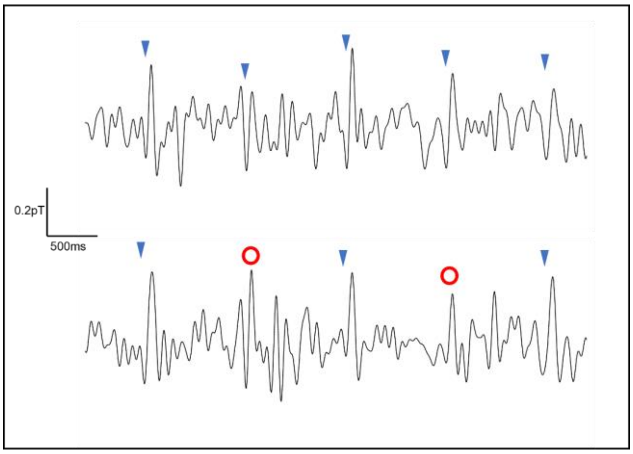

Supplementary Figure 5: Magnetic signals after Low-pass filtering. Representative waveforms are shown after processing the artificial signal data (1.0×) through Low-pass filters with 12Hz. Blue arrows indicate locations where the LSTM network successfully detected peak regions, and red circles indicate locations where peak regions failed to be detected.

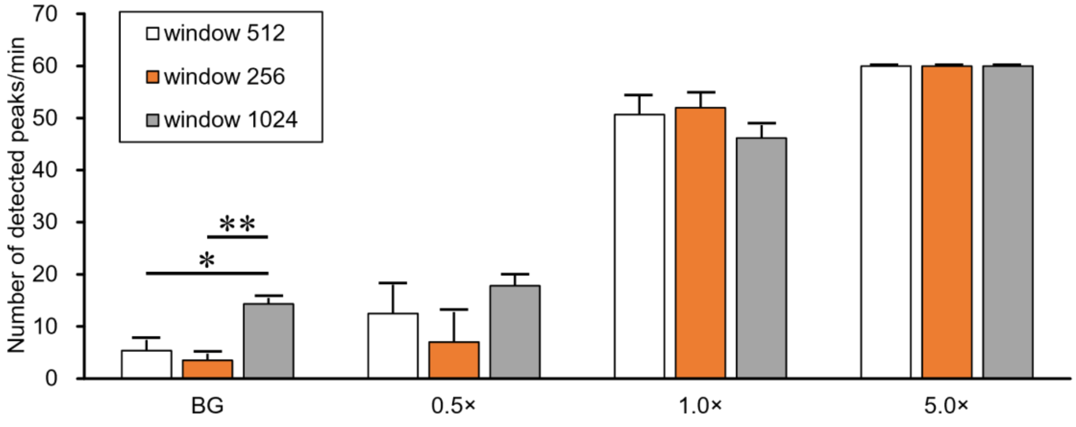

Supplementary Figure 6: Effect of the width of Kaiser window on peak region detection. We compared the number of detected peaks from artificially generated magnetic signals using networks trained with the width of Kaiser window set to 512, 256, and 1024, respectively. The horizontal axis is the intensity of the artificially generated magnetic signals. * $p < 0.05$, ** $p < 0.01$.

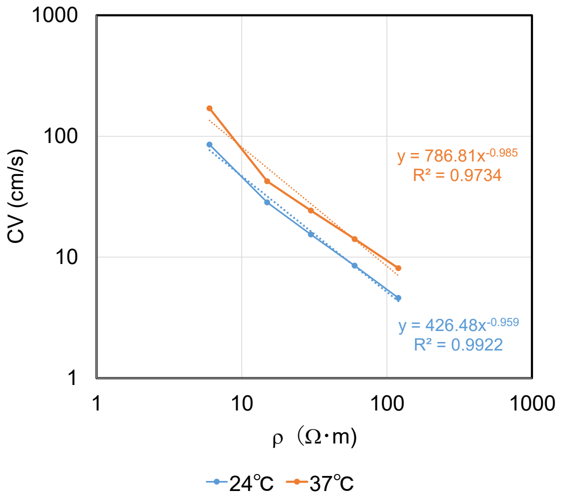

Supplementary Figure 7: Relationship between averaged cellular resistivity and conduction velocity. The conduction velocity estimated from the numerical simulation is plotted logarithmically against averaged cellular resistivity, which is set as a variable parameter. The red line is the result at 37°C, and the blue line is at 24°C (room temperature). The dashed lines in the graph are approximate curves.

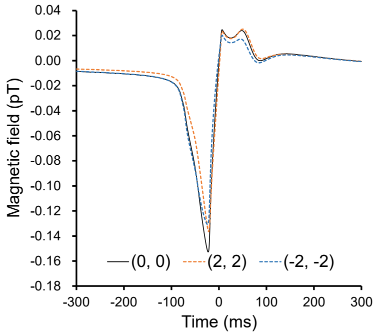

Supplementary Figure 8: Changes in the estimated magnetic fields caused by displacement of cells. The three estimated peak waveforms are plotted overlaid when the center of magnetometer and cells are coincident (black lines), cells are displaced by 2 mm in both the x and y directions (red dotted lines), and by −2 mm (blue dotted lines). The effect of displacement on the estimated waveforms is negligible.

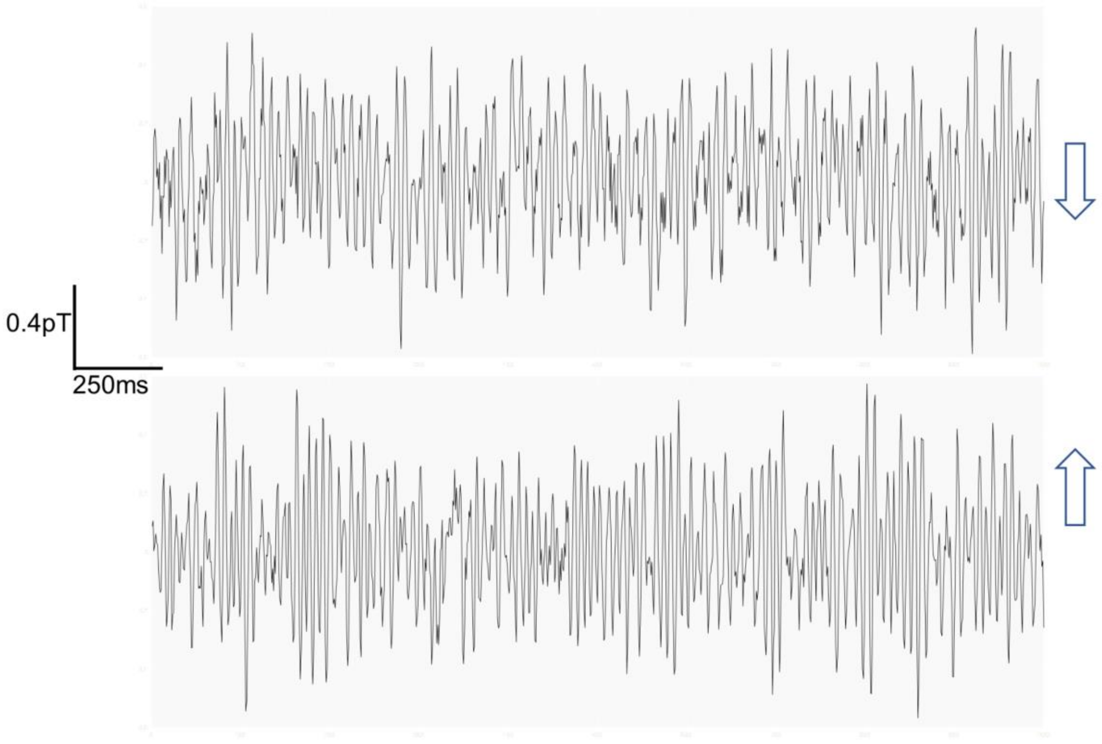

Supplementary Figure 9: Representative waveforms of the data used to train the network for cell experiments are shown. We estimated the magnetic signals from iPS-CMs using numerical simulation and combined them with the *y*-direction component of background noise. Each arrow indicates the direction of the peak.

Supplementary Tables

Supplementary Table 1: Scaling factors for pacemaker-like cell AP model

Current	Scaling
s	0.864
Na	1.652
h	0.202
K	1.332
1	0.154

Note: Optimized scaling factors for five conductances: slow inward current (G_s), sodium current (G_{Na}), delayed inward current activated by hyperpolarization (G_h), potassium current (G_K), and time-independent leak current (G_1).

Supplementary Table 2: Peak detection results for artificially generated magnetic signals

	0.5 ×											
	#1				#2				#3			
	correct	undet	mis		correct	undet	mis		correct	undet	mis	
[3.5, 12]	6	114	1	7	16	104	7	23	38	82	7	45
[3.5, 20]	19	101	12	31	24	96	12	36	42	78	18	60
[3.5, 40]	32	88	27	59	33	87	32	65	53	67	33	86
[12, 20]	2	118	2	4	0	120	8	8	2	118	6	8
noBG	85	35	89	174	99	21	105	204	101	19	78	179
	1.0 ×											
	#4				#5				#6			
	correct	undet	mis		correct	undet	mis		correct	undet	mis	
[3.5, 12]	90	30	7	97	91	29	1	92	107	13	8	115
[3.5, 20]	84	36	12	96	87	33	6	93	90	30	14	104
[3.5, 40]	91	29	12	103	79	41	20	99	92	28	21	113
[12, 20]	4	116	2	6	5	115	3	8	8	112	4	12
noBG	113	7	82	195	111	9	86	197	112	8	80	192
	5.0 ×											
	#7				#8				#9			
	correct	undet	mis		correct	undet	mis		correct	undet	mis	
[3.5, 12]	120	0	0	120	120	0	0	120	120	0	0	120
[3.5, 20]	120	0	0	120	118	2	0	118	120	0	2	122
[3.5, 40]	120	0	2	122	120	0	0	120	118	2	1	119
[12, 20]	73	47	1	74	67	53	0	67	76	44	0	76
noBG	120	0	16	136	120	0	11	131	120	0	15	135

Note: Number of peaks detected from 120 seconds of data. The leftmost column shows the frequency range used for training the network. The numbers highlighted in gray are peak detections using the network (the sum of correct detections and mis-detections).

Supplementary Table 3: Constants and initial conditions used in the 2D simulation

Parameter	Definition	Value
T	Temperature	297 K
V_C	Cytoplasmic volume	2587.37 μm^3
V_{SR}	Sarcoplasmic reticulum volume	171.63 μm^3
C_m	Cell Capacitance	18.50 pF
S	Surface-to-volume ratio	0.1 μm^{-1}
ρ	Cellular average resistivity	60 Ω·m
V_m	Membrane potential	−70 mV
$[K^+]_o$	Extracellular K^+ concentration	4.4 mM
$[Na^+]_o$	Extracellular Na^+ concentration	115.8 mM
$[Ca^{2+}]_o$	Extracellular Ca^{2+} concentration	1.5 mM
$[K^+]_i$	Intracellular K^+ concentration	143.72 mM
$[Na^+]_i$	Intracellular Na^+ concentration	14.2371 mM
$[Ca^{2+}]_i$	Intracellular Ca^{2+} concentration	0.115001 μM
$[Ca^{2+}]_{SR}$	Sarcoplasmic reticulum Ca^{2+} concentration	1.2995 mM

References

1. Bot, C. T., Kherlopian, A. R., Ortega, F. A., Christini, D. J. & Krogh-Madsen, T. Rapid genetic algorithm optimization of a mouse computational model: Benefits for anthropomorphization of neonatal mouse cardiomyocytes. *Front. Physiol.* **3 NOV**, 1–14 (2012).
2. Deb, K. & Agrawal, R. B. Simulated Binary Crossover for Continuous Search Space. *Complex Syst.* **9**, (1995).
3. Deb, K. & Deb, D. Analysing Mutation Schemes for Real-Parameter Genetic Algorithms. *Int. J. Artif. Intell. Soft Comput.* **4**, 1–28 (2014).
4. Kadota, S. *et al.* Development of a reentrant arrhythmia model in human pluripotent stem cell-derived cardiac cell sheets. *Eur. Heart J.* **34**, 1147–1156 (2013).
